# Predicting improvement in biofeedback gait training using short-term spectral features from minimum foot clearance data

**DOI:** 10.3389/fbioe.2024.1417497

**Published:** 2024-08-28

**Authors:** Nandini Sengupta, Rezaul Begg, Aravinda S. Rao, Soheil Bajelan, Catherine M. Said, Marimuthu Palaniswami

**Affiliations:** ^1^ Department of Electrical and Electronic Engineering, The University of Melbourne, Parkville, VIC, Australia; ^2^ Institute for Health and Sport, Victoria University, Melbourne, VIC, Australia; ^3^ Physiotherapy, Melbourne School of Health Sciences, The University of Melbourne, Parkville, VIC, Australia; ^4^ Physiotherapy Department, Western Health, St Albans, VIC, Australia; ^5^ Australian Institute for Musculoskeletal Science (AIMSS), Melbourne, VIC, Australia; ^6^ Physiotherapy Department, Austin Health, Heidelberg, VIC, Australia

**Keywords:** stroke rehabilitation, biofeedback, treadmill training, interventions, machine learning, signal processing

## Abstract

Stroke rehabilitation interventions require multiple training sessions and repeated assessments to evaluate the improvements from training. Biofeedback-based treadmill training often involves 10 or more sessions to determine its effectiveness. The training and assessment process incurs time, labor, and cost to determine whether the training produces positive outcomes. Predicting the effectiveness of gait training based on baseline minimum foot clearance (MFC) data would be highly beneficial, potentially saving resources, costs, and patient time. This work proposes novel features using the Short-term Fourier Transform (STFT)-based magnitude spectrum of MFC data to predict the effectiveness of biofeedback training. This approach enables tracking non-stationary dynamics and capturing stride-to-stride MFC value fluctuations, providing a compact representation for efficient processing compared to time-domain analysis alone. The proposed STFT-based features outperform existing wavelet, histogram, and Poincaré-based features with a maximum accuracy of 95%, F1 score of 96%, sensitivity of 93.33% and specificity of 100%. The proposed features are also statistically significant (*p* < 0.001) compared to the descriptive statistical features extracted from the MFC series and the tone and entropy features extracted from the MFC percentage index series. The study found that short-term spectral components and the windowed mean value (DC value) possess predictive capabilities regarding the success of biofeedback training. The higher spectral amplitude and lower variance in the lower frequency zone indicate lower chances of improvement, while the lower spectral amplitude and higher variance indicate higher chances of improvement.

## 1 Introduction

Stroke affects millions of people worldwide each year (Gerstl et al., 2023) and approximately 60,000 in Australia, i.e., more than 100 documented incidents daily.^1^ Stroke is a prevalent and significant health risk associated with ageing, with stroke patients often exhibiting impaired gait dynamics of varying severity. Stroke survivors with impaired gait dynamics commonly experience a higher likelihood of falls (Roelofs et al., 2023). Minimum Foot Clearance (MFC) is the foot’s minimum vertical displacement from the walking surface during the mid-swing phase of the walking cycle. Low MFC can increase the risk of tripping-related falls (Nagano et al., 2020; Nagano et al., 2022). Assessing the effectiveness of gait training requires multiple training sessions, with follow-up clinical evaluations requiring major resources. Fall prevention programs based on exercises have proven beneficial for the general older adult population, but they lack effectiveness when applied to stroke-injured individuals (Begg et al., 2019). In one falls intervention study of stroke patients, a home-based balance and strength program was trialled (Batchelor et al., 2012), and in another, an exercise program with both group and home-based balance and strength training was conducted (Dean et al., 2012), but neither demonstrated a reduction in falls.

**FIGURE 1:**
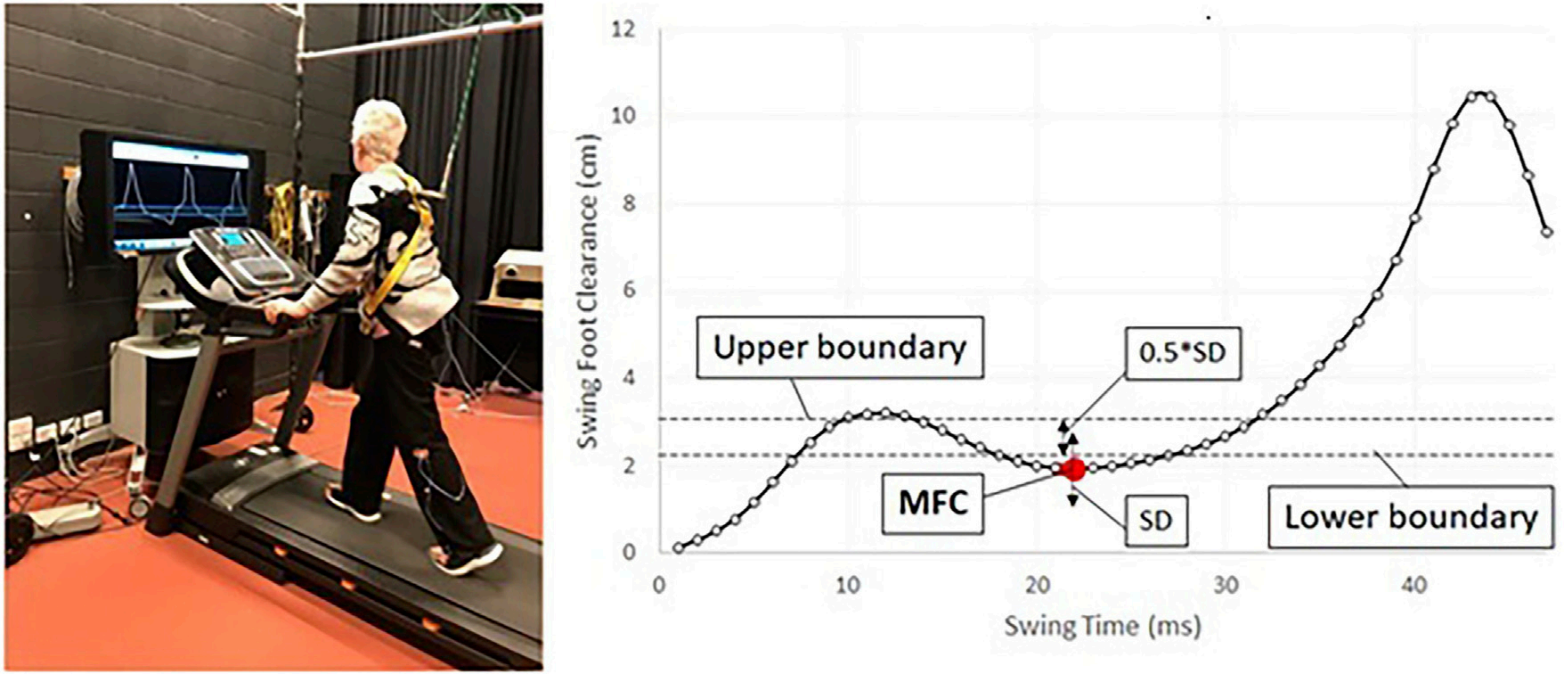
The diagram depicts a person engaged in treadmill training while receiving real-time biofeedback. The training aims to regulate minimum foot clearance (MFC) data within a target band, indicated by a red dot. The target band is defined by the (mean + SD) ± (0.5*SD) (standard deviation) of MFC, establishing the upper and lower boundaries (Nagano et al., 2022). Adapted from Nagano et al. (2022), licensed under CC-BY 4.0 and the authors have made no changes to the original figure.

These traditional stroke rehabilitation methods are hindered by the absence of real-time, objective feedback, limiting patient engagement and impeding effective progress (Teodoro et al., 2024; Spencer et al., 2021; Giggins et al., 2013). To overcome the limitations of traditional stroke rehabilitation, our research group has pioneered treadmill-based biofeedback training (Begg et al., 2014) by presenting a real-time display of the forefoot marker’s trajectory on a video monitor positioned in front of the treadmill (Begg et al., 2019; van der Straaten et al., 2020). Figure 1 shows an example of real-time biofeedback treadmill training and the associated MFC series. The principal biofeedback variable derived from the forefoot marker is the MFC (see Figure 1) (Nagano et al., 2022). MFC at mid-swing is the critical gait variable in predicting tripping (Nagano et al., 2022; Begg et al., 2019) with low MFC leading to unanticipated, destabilizing, foot-ground contacts (Pathak et al., 2022; Best and Begg, 2008). Stroke participants who have difficulty in stepping over relatively low surface irregularities of approximately 4 cm are at increased risk of falling (Said et al., 2014), and they often exhibit lower and more variable MFC control across multiple steps (Pathak et al., 2022).

Biofeedback gait training has been shown to be effective in stroke rehabilitation to improve MFC data by controlling swing foot movements (Begg et al., 2014; Nagano et al., 2022), where people can receive real-time visual feedback to control MFC within the target band, determined by individuals’ swing foot motions (Nagano et al., 2022). A uniform rehabilitation program may not be suitable for all individuals, and the capacity to predict the effectiveness of gait training from pre-intervention MFC data would be highly beneficial, potentially saving public health costs and reducing patient inconvenience. The aim of this project was to predict the effectiveness of biofeedback treadmill training for stroke patients from their baseline walking data before training. Gait improvements are identified from increased MFC within an individual-specific threshold. We hypothesized that biofeedback training effects on MFC could be predicted using novel features of the Short-Term Fourier Transform (STFT) magnitude spectrum of MFC data. This approach was expected to enable the tracking of non-stationary dynamics and capturing stride-to-stride MFC fluctuations (Pachori, 2023), providing compact representations and more efficient processing than time-domain analysis alone.

Our main contributions to this work are threefold:

• We propose new STFT-based magnitude spectrum features derived from MFC data to predict the effectiveness of biofeedback gait training. The mean short-term magnitude spectrum reveals the tendency of stride-to-stride fluctuation in MFC values, providing insights into gait stability. Analyzing frequency-related information helps identify patterns and irregularities in movement across strides. To the best of our knowledge, this is the first study to propose short-term spectral features to predict improvement in biofeedback gait training from baseline data. Although histogram and Poincaré features (Begg et al., 2007) and wavelet-based features (Khandoker et al., 2007) were applied to recognise gait patterns, these methods were used primarily in the context of healthy adults, and their performance in the analysis of MFC data in stroke patients remains unknown.

• MFC data has dynamic patterns and is non-stationary. To enable tracking of non-stationary MFC dynamics and to capture the patterns, we first decompose the baseline MFC series into their underlying sinusoidal structures, revealing embedded stride-to-stride fluctuations. Then, we make use of three frequency components derived from STFT-based magnitude spectrum features to predict biofeedback-based training from baseline MFC data. The three derived frequency components demonstrate high statistical significance (p<0.001) , making our proposed framework superior to existing approaches.

• We provide a comprehensive analysis of spectral features and evaluate these components in combination with machine-learning classification models to develop predictions from baseline data. In addition, we provide an analysis of windowing the MFC series and the robustness of frequency components under noisy conditions.

## 2 Related work

Previous studies have primarily focused on analyzing the linear statistical properties of biomechanical variables to investigate safer walking and lower-limb control characteristics (Khandoker et al., 2016; Begg et al., 2005). Statistical features, including mean, standard deviation (s.d.), skewness, kurtosis, median, 25th and 75th percentiles, interquartile range, mode, minimum, maximum, and quartile coefficient of dispersion were extracted (Begg et al., 2005). They also utilized Poincaré plots to visually represent the relationship between successive gait cycles and provide insights into the performance of the locomotor system in controlling critical events. From the Poincaré plot, they extracted features corresponding to both the major and minor axes, capturing short- and long-term variability in the MFC data. The other research work (Khandoker et al., 2016) also analyzed descriptive statistics to quantify the MFC series, including the mean, median, standard deviation (SD), 25th percentile (Q1), 75th percentile (Q3), and interquartile range (IQR). In addition, the authors also introduced tone and entropy features based on the percentage change in successive MFC observations relative to the previous MFC, referred to as the Percentage Index (PI).

To consider the complexity and nonstationary properties of the MFC series, a wavelet-based multiscale exponent to capture correlations among the variances of wavelet coefficients across different scales was employed (Khandoker et al., 2007). The MFC series underwent decomposition using Dabaucheis wavelets of order 6, with eight levels of decomposition, resulting in a sequential list of detailed coefficients that represented the correlation evolution between the series and selected frequencies within various frequency ranges. Although these methods have shown success in healthy adults, their performance in analysing MFC data in stroke patients remains unknown.

Short-term magnitude spectrum is valuable for observing fluctuations in MFC values in consecutive strides within smaller intervals. Its analysis of stride-to-stride fluctuations provides insights into gait characteristics, specifically regarding the consistency and stability of MFC fluctuations across strides. This information is crucial for assessing mobility and functional recovery in stroke patients, as it might reflect gait stability and muscle coordination.

## 3 Materials and method

**FIGURE 2:**
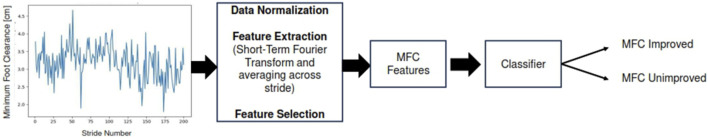
A high-level overview of the proposed approach. Baseline MFC data is processed to extract MFC features and then fed to a classifier to predict whether there is an improvement in the MFC or not.

The MFC series offers valuable insights into foot trajectory control. The short-term average is suitable for characterizing MFC control in stroke patients because it represents the overall intensity or strength of the MFC signal within short intervals and accommodates nonstationary characteristics of the MFC series (Khandoker et al., 2007). Reduced MFC fluctuation suggests a more stable gait; in such cases, the short-term average can effectively capture the overall intensity of the MFC signal. This measure is particularly useful for quantifying the stability and regularity of gait in stroke patients due to the focus on average signal magnitude rather than time-frequency characteristics. While post-training assessments are typically used to determine any improvements during stroke rehabilitation, our objective here was to *predict* training effects. This is a novel problem in stroke rehabilitation and this report is the first to address this problem, Figure 2 illustrates our approach.

### 3.1 Participants

This study included 19 patients over 18 years of age at least 6 months after a single stroke (ischemic or hemorrhagic). They could walk independently for 50 m and were able to provide informed consent (Begg et al., 2019). Patients were excluded if they had an ankle orthosis, any other medical condition that prevented them from walking on a treadmill, visual deficits, or body mass exceeding 158 kg (Begg et al., 2019), participant characteristics have been presented in Table 1. Participants were carefully briefed and their consent was secured to ensure informed participation. The study was included in the Australian and New Zealand Clinical Trials Registry - trial ACTRN12617000250336 and approved by the Human Research Ethics Committees of Victoria University, Australia and Austin Hospital, Melbourne, Australia.

**TABLE 1:** The table summarizes the participants’ details, including the number of subjects, age, affected lower limb, and walking speed among 19 subjects.

Class	Improved	Unimproved
No. of subjects	14	5
Age (years)	68.71±12.31	69.40±11.76
Female	6	1
Left affected	5	5
Walking speed (km/h)	1.96±1.04	2.08±0.48

### 3.2 Data collection

We employed a three-dimensional motion analysis system (Optotrak^®^, NDI, Canada) to capture kinematic data at 100 Hz. Following a standardized protocol (Begg et al., 2007), participants were outfitted with a cluster of three active markers, including one affixed to the big toe. The forefoot’s imaginary position was digitized using an active digitizing probe. To ensure safety and adherence to the protocol, all participants were secured by a safety harness and instructed to walk on a motorized treadmill at their self-selected walking speed for up to 10 min, with rest breaks as needed. During subsequent biofeedback gait training sessions, the real-time sagittal trajectory of the big toe marker was displayed on a screen positioned in front of the treadmill (see Figure 1). This display featured toe clearance, associated MFC events and the individual patient’s training-target MFC from their baseline MFC data, depicted as a horizontal line on the screen (Begg et al., 2019). Participants were then tasked with adjusting their MFC height to match the monitored range. Patients underwent a total of 10 biofeedback training sessions, with faded biofeedback introduced after the initial six sessions. Detailed information about the biofeedback training sessions is available in Begg et al. (2019).

### 3.3 Assessment

Gait assessment tests were scheduled at the baseline and immediately after the final training session (with a minimum gap of 20 min). The class labels were based on post-training MFC change from baseline MFC data by which participants could be categorized as either improved or unimproved following training.

### 3.4 Spectral analysis of baseline MFC data

The short-term average provides the overall intensity or strength of the MFC signal within short intervals, which covers the nonstationary characteristics of the MFC series.

The height of the MFC refers to the vertical displacement between the lowest point of the foot (represented by the toe marker) and the ground during the swing phase of walking (Begg et al., 2007). Then the series of the MFC height can be represented as in Equation 1

$$MFC=\left\{MFC_{1},MFC_{2},MFC_{3},\ldots ,MFC_{N}\right\},$$ (1)

where N is the number of MFC data points (Khandoker et al., 2016). We normalize the MFC series to reduce the effects of lengthy series and between-subject variability.

We can now define the STFT of the MFC series as in Equation 2

$$MFC_{f}\left[k,m\right]=\overset{N-1}{\underset{n=0}{\sum }}MFC\left[n\right]w\left[n-m\right]e^{-j\frac{2\pi }{N}kn},$$ (2)

where w[n] is the window function, m is the shift parameter, k is the frequency bin index, and N is the length of the signal.

The average magnitude across frames can be calculated as in Equation 3

$$MFC_{f}\left(k\right)=\overset{L-1}{\underset{m=0}{\sum }}\frac{\left|MFC_{f}\left(k,m\right)\right|}{L},$$ (3)

where L is the number of frames.

**FIGURE 3:**
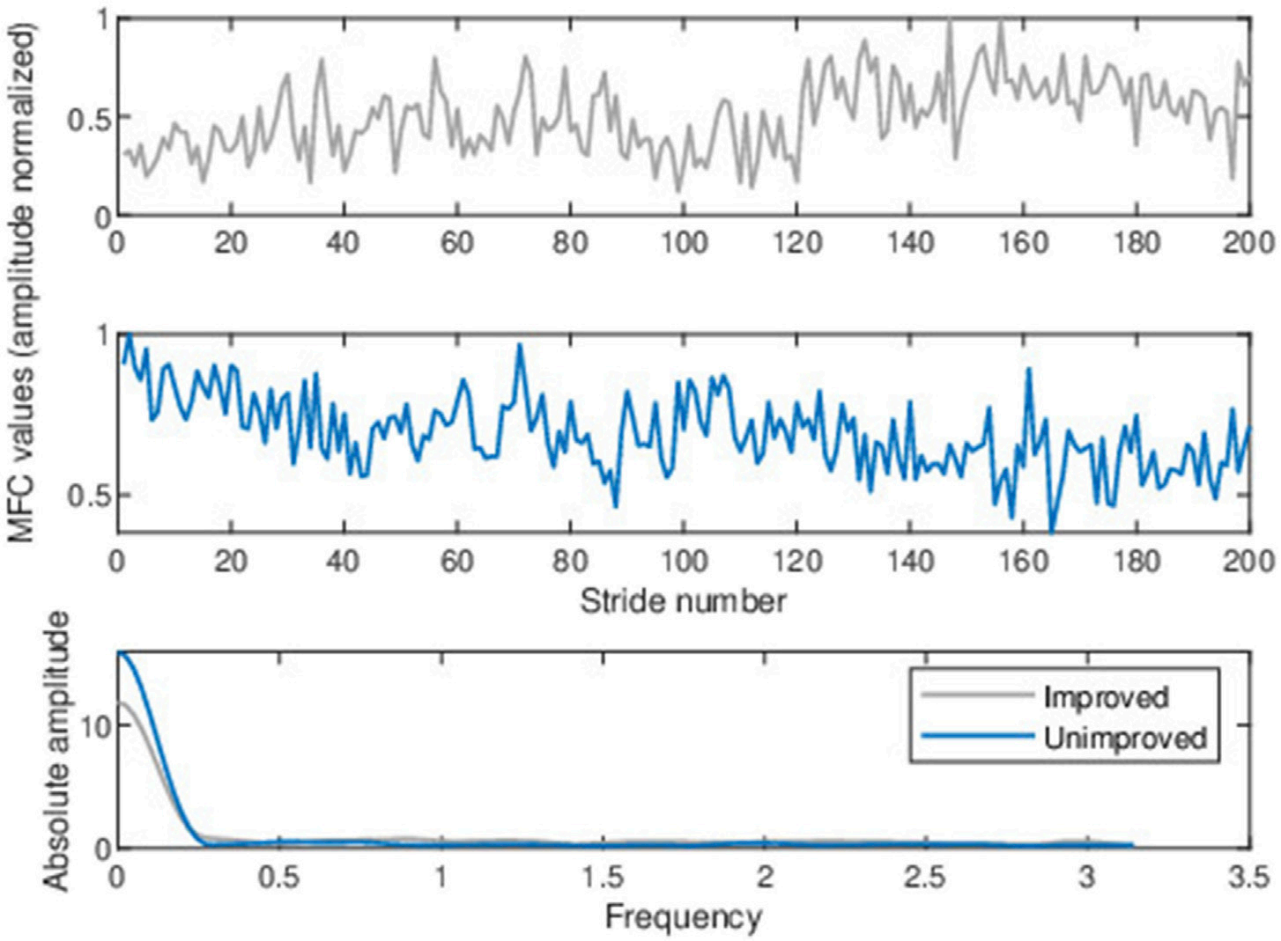
Typical baseline normalized MFC height series for an arbitrarily chosen stroke patient (upper panel) who improved after training and another patient who did not improve after training (middle panel). The magnitude (absolute amplitude) spectrum was computed from the normalized baseline MFC data above (lower panel).

**FIGURE 4:**
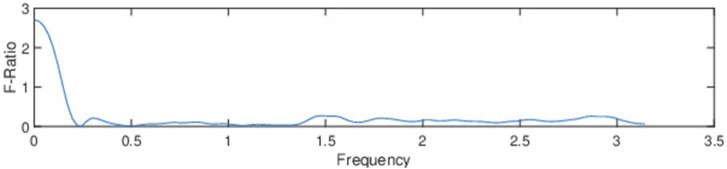
The F-ratio of magnitude-spectrum coefficients demonstrates the separability between improved and unimproved.

Figure 3 file shows the MFC series for improved and unimproved patients with their frequency domain amplitude spectrum below. From Figure 3, it is evident that the lower frequency range appears to exhibit notable discriminant characteristics, but we can employ F-tests to more reliably confirm the separability of data (Nicholson et al., 1997; Sengupta et al., 2016). The F statistic is the ratio of the between-class and within-class variance of magnitude spectrum coefficients for a particular frequency component. Figure 4 shows the F-ratio plot, with a higher F-ratio indicating more separation between the classes. For a given frequency component k , the F-ratio is defined as in Equation 4

$$F\left(k\right)=\frac{\sum_{i=1}^{C}{\left[{\bar{S}}_{i}\left(k\right)-\bar{S}\left(k\right)\right]}^{2}}{\sum_{i=1}^{C}\left(\frac{1}{C_{i}}\sum_{j=1}^{C_{i}}{\left[S_{\text{i}}^{\text{j}}\left(k\right)-{\bar{S}}_{i}\left(k\right)\right]}^{2}\right)}$$ (4)

where $S_{\text{i}}^{\text{j}}\left(k\right)$ is the magnitude spectrum coefficient of the j -th sample $\left(j=1,2,\ldots ,C_{i}\right)$ of the i -th class (i=1,2,…,C) at frequency k. ${\bar{S}}_{i}\left(k\right)$ and $\bar{S}\left(k\right)$ are the mean of the k -th frequency component of the i -th class and all the classes, respectively. We calculated F-ratios for different frequency components to analyze the separability of classes in detail. This F-ratio analysis involved using all the MFC series belonging to the two classes. The variation of the F-ratio concerning frequency is depicted in Figure 4. The short-term magnitude spectrum of each MFC series was obtained by dividing it into eight segments with 50% overlap using a Hamming window.

The primary finding from Figure 4 is that the short-term magnitude spectrum in the lowest-frequency region (i.e., 0 to ≈ 0.2 cycles/stride) carries more discriminative information compared to higher-frequency regions. While there are non-zero data of F-ratio in the higher frequency range as well, they are lower than those in the lower frequency zone. Therefore, it is quite evident that the lower frequency zone is more discriminative than the higher frequency zone. The MFC data represents a discrete series since it is generated at specific time points within the strides. Considering that each patient maintained a self-preferred consistent speed on the treadmill, the occurrence of MFC values at equal time intervals enables us to define the frequency as cycles per stride. Our primary focus is on observing the changes in MFC values within each stride, rather than the time gap between consecutive values. To facilitate this analysis, we employed a default normalized sampling frequency, where the term ‘frequency’ refers to a normalized measure.

#### 3.4.1 Spectral feature extraction

**FIGURE 5:**
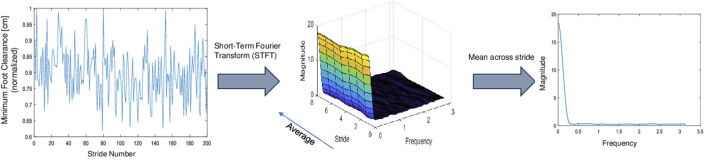
The figure showcases the computation of spectral features from the MFC series through the Short-Term Fourier Transform (STFT). This yields a three-dimensional matrix that captures stride-frames, frequency, and magnitude. Averaging across stride frames provides the dominant frequency component across strides.

The feature calculation involved dividing each MFC series into eight segments with 50% overlap and applying the Hamming window. A 256-point Fast Fourier Transform (FFT) was performed, and by averaging the magnitude over stride frames, we obtained 129 frequency components (including the 0th frequency bin) representing magnitude values at different frequencies. Figure 5 represents a basic diagram of the feature extraction technique comprised of an STFT of the MFC series and then averaging across the stride axis to obtain the feature vector.

We chose the short-term magnitude corresponding to the lowest frequencies as our feature since it demonstrated the maximum differentiation between the two groups as observed in Figures 3, 4 and can be computed as $MFC_{f}\left(k\right)$ . In the following section, we consider the lower frequency zone as our main focus for feature selection because of its superior discrimination power and prominent amplitude values.

### 3.5 Feature significance and selection

To determine the statistical significance of the spectral characteristics, we conducted a Mann-Whitney U-Test (Gibbons and Chakraborti, 2020) on each characteristic of the two groups. For our sample (n = 19) nonparametric estimation was preferred with p <0.05 a suitable threshold for significance. In addition, we computed the AUC values for each feature to investigate their importance further. ROC curves aid in developing an automatic classification model by assessing the effectiveness of each feature at various thresholds (Fawcett, 2004). They highlight the feature’s ability to maximize detection probability while minimizing false alarms. AUC was calculated from the ROC curve for individual spectral characteristics, and a support vector machine (SVM) was used for this binary classification as it showed consistent and reliable performance in the prior studies (Begg et al., 2005; Khandoker et al., 2007). Given our smaller sample size, we adopted a precaution against overfitting by selecting only the three most significant features for classification, balancing the model’s complexity and generalizability (Murphy, 2012).

### 3.6 Predicting the improvement of MFC

To predict the improvement in MFC data from baseline treadmill training, we use five classifiers based on previous literature specifically used in binary classification (Caruana and Niculescu-Mizil, 2006) and our work in this area (Begg et al., 2005; Khandoker et al., 2007). These classifiers include Support Vector Machine (SVM), Random Forest (RF), AdaBoost, Ensemble Decision Tree (EDT) and Artificial Neural Network (ANN).

Support Vector Machine (SVM) is a supervised machine learning technique that is based on guaranteed risk bounds of statistical learning theory known as structural risk minimization (SRM) principle (Burges, 1998) and it is used for both classification and regression. The main function of SVM is to find an optimal hyperplane that effectively separates data points into different classes and maximizes the margin between them. The decision function is given by Equation 5

$$f\left(x\right)=\text{sgn}\left(w.x+b\right)=\text{sgn}\left(\overset{M}{\underset{i=1}{\sum }}\left(\alpha_{i}y_{i}K\left(x_{i},x\right)+b\right)\right)$$ (5)

where, f(x) represents the decision function, w is the weight vector perpendicular to the separating hyperplane, b serves as a bias determining the position of the hyperplane, $x_{i}$ represents the i -th feature vector of dimension d , $y_{i}\in \left\{+1,-1\right\}$ is the label (target output) of $x_{i}$ , $\alpha_{i}$ is the Lagrange multiplier of the i -th data point, $K\left(x_{i},x\right)$ is the kernel function, and M represents the number of *support vectors*—data points in the margin. The sgn(⋅) function returns the sign of the argument.

Kernel techniques facilitate class separation by projecting data points into a high-dimensional space when they are not separable in the lower-dimensional space. Nonlinear kernels, including polynomial kernels, radial basis functions employed in addition to linear kernels (Burges, 1998).

Decision Tree is a non-parametric supervised learning method utilized for both classification and regression tasks (Mitchell, 1997). It uses tree-like structure to make decisions or predictions based on input features. It recursively partitions the data based on feature values, creating a hierarchical structure of decision nodes and leaf nodes. Each internal node represents a decision based on a specific feature, while each leaf node represents a class label.

1. Random Forest (RF) combines multiple Decision Trees through the use of *bagging*, i.e., training each tree on a random subset of the data and considering only a random subset of features at each split (Breiman, 2001). The outcome is determined by averaging or majority voting on the predictions generated by these trees.
2. AdaBoost, short for Adaptive Boosting, is a boosting algorithm that sequentially combines multiple weak learners, often Decision Trees with only one level of depth or “stumps” (Freund and Schapire, 1997). Each weak learner is trained on a weighted version of the training data, with higher weights assigned to misclassified samples. The subsequent weak learners focus more on the previously misclassified samples, improving the overall performance. AdaBoost iteratively updates the sample weights and combines the weak learners’ predictions through weighted voting (Freund and Schapire, 1997).
3. Ensemble Decision Tree (EDT) with *bagging*, combines multiple Decision Trees trained on different bootstrap samples of the training data. Bagging aims to reduce variance and enhance stability by introducing randomness in the training process. Each tree in the ensemble is constructed independently on a randomly drawn subset of the training data with replacement. The final prediction is obtained by aggregating the predictions of all the individual trees, typically through majority voting or averaging (Dietterich, 2000).

Artificial Neural Networks (ANNs), are computational models inspired by the structure and function of the human brain. They consist of artificial neurons that mimic biological neurons and are connected through synapse-like links. ANNs are organized in layers with connections between them. The input layer receives the data to be modelled, and the output layer produces the predicted output. The response produced in the output node is defined as Equation 6:

$$o_{k}=f_{o}\left(\overset{m}{\underset{j=0}{\sum }}w_{k,j}h_{j}\right),$$ (6)

where $o_{k}$ is the produced response of the k -th node of the output layer, $f_{o}$ is the non-linear function at the output layer node, m is the number of nodes in the hidden layer, $w_{k,j}$ is the weight connecting the j -th hidden node and the k -th output node, and $h_{0}=1$ is the bias term. ANN is a supervised classifier, and the weights are determined during the training phase. The backpropagation algorithm and mathematical optimization techniques are used to learn the weights of the connections between neurons, minimizing the difference between the expected and predicted outputs. Learning the proper weights is crucial, and optimization algorithms adjust these weights through mathematical procedures. ANNs can be used for both classification and regression problems (Haykin, 2009).

### 3.7 Model training and evaluation

We used the proposed STFT-based features to develop an automated classification model to identify individuals who would experience improvement based on their baseline MFC series as a result of biofeedback training. Due to limited samples, with five samples in the unimproved class (see Table 1), we approached the cross-validation in three ways:

1. Leave-one-sample-out cross-validation. Each of the 19 samples was taken individually as a test sample, while the remaining samples were used for training. This process was repeated for all 19 samples and the average performance metric was calculated across all samples.
2. Leave-one-fold-out cross-validation. By randomly selecting four samples from each class, we trained the model with these samples while using the remaining samples for testing. This random selection process was repeated 50 times to ensure reliable results. We calculated the average performance metrics for these iterations.
3. 5-fold stratified cross-validation. The data set was divided into five folds and the model was trained in four folds, while one-fold was reserved for testing. This process was repeated five times, ensuring that each fold maintained the original dataset’s class distribution. This approach allowed for a proper proportion of samples from each class in both training and testing sets. Following a 5-fold cross-validation procedure, we calculated the average performance metrics. This involved evaluating the model’s performance in multiple iterations to ensure robustness and reliability.

To ensure generalization accuracy (ACC), this study used three metrics. These metrics, including sensitivity (SENS), specificity (SPEC), and the F1 score (F1), were calculated for each class in all subjects (Murphy, 2012). This validation method allowed evaluation of the model’s performance while accounting for variation between subjects and ensuring the ability to generalize to unseen data.

## 4 Results

We present our results and analyses in five subsections highlighting the significance of spectral features, including statistical significance of the spectral features, comparison with other MFC features, comparison of features with alternative classifiers, MFC window effects and performance under noisy conditions.

### 4.1 Statistical significance of the spectral features

**FIGURE 6:**
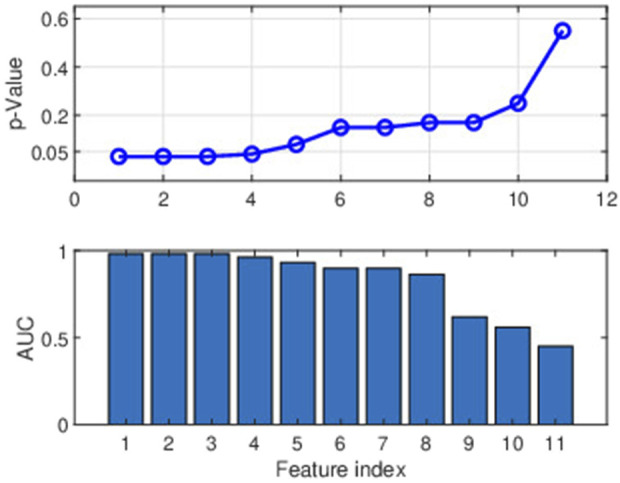
The figure depicts the two parameters (*p*-value and AUC) for selecting the spectral features. The upper panel represents the significance of 11 features across lower frequencies using *p*-values. The first 11 features consist of the DC value and the absolute amplitudes (magnitudes) corresponding to the first 10 frequencies (frequency range of 0–0.2545 cycles/stride). The lower panel shows the efficacy of modelling individual characteristics regarding AUC values.

The MFC frequency spectrum in Figure 3 indicates that the lower frequency range exhibited greater discrimination between improved and non-improved classes, with differentiation declining as frequency increments. The same trend is seen in Figure 6, in which the first 11 spectral components are presented with Mann Whitney *p*-values and AUC statistics from the SVM classifier (refer to Section 3.5). The consistent findings are shown in Figures 3, 6 provide strong evidence that the lower frequency range contains the most valuable discriminant features.

The leave-one-fold-out cross-validation (refer to Section 3.7 second approach) showed that the *p*-values of the first three features [ $MFC_{f}\left(0\right)$ , $MFC_{f}\left(1\right)$ and $MFC_{f}\left(2\right)$ ] were 0.03(<0.05) , indicating strong statistical significance, i.e., below the 0.05 threshold, but *p*-values exceeded 0.05 after the fourth feature. The initial three values contributed to approximately 0.98 AUC but subsequently decreased.

We used statistical analysis to compare the results, similar to Khandoker et al. (2016). We calculated the following descriptive statistics to quantify the MFC series: mean, median, standard deviation (SD), Q1 (25th percentile), Q3 (75th percentile), IQR (Q1-Q3, interquartile range); additionally, we used tone and entropy estimates from the MFC Percentage Index (PI) series (Khandoker et al., 2016). The Mann-Whitney U test (Gibbons and Chakraborti, 2020) was used to determine statistically reliable differences in these statistics between the two groups of stroke patients, that is, improved and unimproved.

Table 2 displays the mean and standard deviation (std) values for all the features mentioned in Khandoker et al. (2016), as well as our proposed feature for two groups: improved and unimproved. On examination, it is evident that all features, except the entropy feature, demonstrated statistical significance when considering a threshold of p<0.05 . However, the proposed spectral feature stood out as it remained significant even when considering a more stringent threshold of p<0.001 (Singh, 2013). This highlights the notable significance and efficacy of our proposed feature (all three spectral features) in effectively distinguishing between the two groups.

**TABLE 2:** Mean ± SD Values of each of three proposed spectral features, tone-entropy parameters, and MFC descriptive statistics (Khandoker et al., 2016) for the improved and unimproved groups of stroke patients.

Feature	Unimproved	Improved	*p*-value
Mean	0.7148 ± 0.0537	0.5164 ± 0.1282	0.005
Median	0.7083 ± 0.0556	0.5068 ± 0.1316	0.007
STD	0.0933 ± 0.0186	0.1351 ± 0.0319	0.014
Q1	0.6492 ± 0.0620	0.4163 ± 0.1373	0.003
Q3	0.7751 ± 0.0472	0.5990 ± 0.1305	0.014
IQR	0.1258 ± 0.0224	0.1827 ± 0.0566	0.055
Tone	−0.9642 ± 0.2923	−5.715 ± 4.1928	0.007
Entropy	2.6811 ± 0.3523	2.6181 ± 0.3399	0.516
Proposed	16.6300 ± 1.2512	11.4983 ± 2.2320	**0.0003**

All statistical comparisons were accepted as significant when *p* < 0.001.

Bold value represents the best performance.

### 4.2 Comparison of MFC-based features

Tables 3–5 present the results of the features previously used in Begg et al. (2005), Khandoker et al. (2007) and our proposed spectral feature evaluated using an SVM classifier. In Begg et al. (2005), the authors used a total of 24 features, including statistical features extracted from the histogram representation of MFC data and features derived from the Poincaré plot of MFC data (Begg et al., 2005). In Khandoker et al. (2007), six features were derived from the MFC values from wavelet decomposition. We chose to use the SVM classifier due to its performance in using the features presented in Begg et al. (2005), Khandoker et al. (2007).

**TABLE 3:** The classification performance of the SVM classifier uses the **leave-one-sample-out cross-validation** method with different kernels (linear, Gaussian RBF, and polynomial) for different regularization parameters (C).

Parameters		Spectral feature					Histogram-Poincaré based feature					Wavelet-based feature				
Kernel	C	ACC	SENS	SPEC	F1	AUC	ACC	SENS	SPEC	F1	AUC	ACC	SENS	SPEC	F1	AUC
Linear	**0.01**	89.47	92.85	80.00	92.85	92.85	73.68	100.00	0.00	84.85	78.57	73.68	100.00	0.00	84.85	35.71
	**0.1**	84.21	85.72	80.00	88.89	94.28	73.68	100.00	0.00	84.85	77.14	73.68	100.00	0.00	84.85	35.71
	**1**	**94.73**	**92.85**	**100**	**96.29**	**94.28**	63.15	85.71	0.00	77.42	82.85	68.42	85.71	20.00	80.00	41.43
	**10**	89.47	92.85	80.00	92.85	92.85	84.21	85.71	80.00	88.89	92.85	63.15	78.57	20.00	75.86	27.14
	**100**	89.47	92.85	80.00	92.85	77.14	84.21	85.71	80.00	88.89	92.85	52.63	64.28	20.00	66.67	20.00
RBF	**0.01**	73.68	100.00	0.00	84.84	82.85	73.68	100.00	0.00	84.85	78.57	73.68	100.00	0.00	84.85	41.43
	**0.1**	73.68	100.00	0.00	84.84	82.85	73.68	100.00	0.00	84.85	80.00	73.68	100.00	0.00	84.85	42.85
	**1**	84.21	92.85	60.00	89.65	84.28	73.68	100.00	0.00	84.85	80.00	73.68	100.00	0.00	84.85	42.85
	**10**	84.21	92.85	60.00	89.65	72.85	63.15	85.71	0.00	77.42	87.14	63.15	78.57	20.00	75.86	48.57
	**100**	68.42	78.57	40.00	78.57	74.28	84.21	85.71	80.00	88.89	92.85	63.15	78.57	20.00	75.86	48.57
Polynomial	**0.01**	89.47	92.85	80.00	92.85	77.14	73.68	100.00	0.00	84.85	84.28	73.68	100.00	0.00	84.85	40.00
	**0.1**	89.47	92.85	80.00	92.85	74.28	73.68	100.00	0.00	84.85	74.28	68.42	92.85	0.00	81.25	41.42
	**1**	89.47	92.85	80.00	92.85	71.43	73.68	100.00	0.00	84.85	77.14	63.15	78.57	20.00	75.86	42.85
	**10**	84.21	85.71	80.00	88.89	87.14	68.42	92.85	0.00	81.25	75.71	52.63	57.14	40.00	64.00	27.14
	**100**	84.21	85.71	80.00	88.89	88.57	78.94	85.71	60.00	85.71	92.85	42.10	42.85	40.00	52.17	25.71

The results reported are for the three spectral features. Best performance is obtained with the spectral feature using the linear kernel (C =1 ): 94.73% Accuracy, 92.85% Sensitivity, 100% Specificity, 96.29% F1-score. (ACC, accuracy; SENS, sensitivity; SPEC, specificity; F1, F1-score; AUC, AREA UNDER the ROC CURVE).

Bold values represent the best performance.

**TABLE 4:** The classification performance of the SVM classifier uses the **leave-one-fold-out cross-validation** method with different kernels (linear, Gaussian RBF, and polynomial) for different regularization parameters (C).

Parameters		Spectral feature					Histogram-Poincaré based feature					Wavelet-based feature				
Kernel	C	ACC	SENS	SPEC	F1	AUC	ACC	SENS	SPEC	F1	AUC	ACC	SENS	SPEC	F1	AUC
Linear	**0.01**	82.91	81.20	100.00	89.40	98.20	33.09	26.40	100.00	37.23	69.60	53.81	57.00	22.00	64.09	33.60
	**0.1**	83.45	81.80	100.00	89.73	98.20	33.81	27.20	100.00	38.56	74.60	54.55	56.80	32.00	66.62	43.40
	**1**	85.64	84.20	100.00	91.11	98.20	62.18	58.40	100.00	71.31	84.20	52.00	52.60	46.00	63.93	51.00
	**10**	85.64	84.80	94.00	91.14	98.20	72.73	70.80	92.00	81.78	83.60	48.90	49.80	40.00	61.43	44.00
	**100**	85.64	84.80	94.00	91.14	98.20	70.00	68.00	90.00	79.51	82.60	48.90	49.60	42.00	61.73	45.20
RBF	**0.01**	78.36	77.60	86.00	85.80	93.40	34.72	28.20	100.00	38.92	81.60	54.72	56.00	42.00	67.17	55.20
	**0.1**	78.36	77.60	86.00	85.80	93.40	34.72	28.20	100.00	38.92	81.60	54.72	56.00	42.00	67.17	55.20
	**1**	78.55	77.80	86.00	85.72	92.20	34.90	28.40	100.00	39.28	81.40	54.00	55.20	42.00	66.37	53.80
	**10**	78.91	78.80	80.00	85.95	88.20	56.91	52.80	98.00	66.48	85.00	48.90	49.40	44.00	61.71	47.40
	**100**	75.45	75.60	74.00	83.50	81.80	71.45	69.00	96.00	80.36	81.60	48.90	49.40	44.00	61.71	47.40
Polynomial	**0.01**	**88.18**	**87.60**	**94.00**	**92.95**	**98.20**	27.45	20.60	96.00	30.26	58.20	68.36	72.80	24.00	71.72	37.40
	**0.1**	87.82	87.20	94.00	92.73	98.00	27.45	20.60	96.00	30.26	62.20	62.00	65.80	24.00	67.89	43.40
	**1**	86.91	86.20	94.00	92.13	97.80	29.64	23.00	96.00	34.14	67.20	48.36	49.80	34.00	59.32	38.20
	**10**	86.18	85.40	94.00	91.65	98.20	36.55	30.60	96.00	44.69	77.00	42.90	43.20	40.00	54.72	36.60
	**100**	86.00	85.20	94.00	91.48	98.20	67.09	65.20	86.00	76.34	80.80	42.91	43.20	40.00	54.72	36.60

The results reported are for the three spectral features. Best performance is obtained with spectral feature in Polynomial kernel (C =0.01 ): 88.18% Accuracy, 87.60% Sensitivity, 94% Specificity, 92.95% F1-score.

Bold value represents the best performance.

**TABLE 5:** The classification performance of the SVM classifier uses the **stratified five-fold cross-validation method** with different kernels (linear, Gaussian RBF, and polynomial) for different regularization parameters (C).

Parameters		Spectral feature					Histogram-Poincaré based feature					Wavelet-based feature				
Kernel	C	ACC	SENS	SPEC	F1	AUC	ACC	SENS	SPEC	F1	AUC	ACC	SENS	SPEC	F1	AUC
Linear	**0.01**	90.00	93.33	80.00	93.14	80.00	73.33	100.00	0.00	84.57	83.33	73.33	100.00	0.00	84.57	33.33
	**0.1**	83.33	83.33	80.00	86.47	80.00	73.33	100.00	0.00	84.57	83.33	73.33	100.00	0.00	84.57	33.33
	**1**	**95.00**	**93.33**	**100.00**	**96.00**	**80.00**	73.33	83.33	40.00	80.76	83.33	75.00	93.33	20.00	84.76	33.33
	**10**	90.00	93.33	80.00	93.14	80.00	78.33	70.00	100.00	79.33	83.33	65.00	80.00	20.00	75.62	26.67
	**100**	90.00	93.33	80.00	93.14	80.00	83.33	76.67	100.00	85.33	83.33	60.00	73.33	20.00	70.28	26.67
RBF	**0.01**	73.33	100.00	0.00	84.57	80.00	73.33	100.00	0.00	84.57	90.00	73.33	100.00	0.00	84.57	46.67
	**0.1**	73.33	100.00	0.00	84.57	80.00	73.33	100.00	0.00	84.57	90.00	73.33	100.00	0.00	84.57	53.33
	**1**	85.00	93.33	60.00	90.28	80.00	73.33	100.00	0.00	84.57	90.00	68.33	93.33	0.00	80.76	53.33
	**10**	85.00	93.33	60.00	90.28	53.33	78.33	90.00	40.00	84.76	90.00	56.67	70.00	20.00	67.62	50.00
	**100**	71.67	73.33	60.00	70.28	53.33	88.33	83.33	100.00	89.33	73.33	56.67	70.00	20.00	67.62	50.00
Polynomial	**0.01**	90.00	93.33	80.00	93.14	80.00	73.33	100.00	0.00	84.57	76.67	73.33	100.00	0.00	84.57	33.33
	**0.1**	90.00	93.33	80.00	93.14	80.00	73.33	100.00	0.00	84.57	83.33	68.33	93.33	0.00	80.76	40.00
	**1**	90.00	93.33	80.00	93.14	80.00	73.33	100.00	0.00	84.57	90.00	70.00	86.67	20.00	80.95	33.33
	**10**	90.00	93.33	80.00	93.14	80.00	73.33	100.00	0.00	84.57	90.00	60.00	66.67	40.00	68.47	13.33
	**100**	90.00	93.33	80.00	93.14	80.00	78.33	83.33	60.00	83.62	83.33	53.33	66.67	20.00	64.47	13.33

The results reported are for the three spectral features. Best performance is obtained with spectral feature in linear kernel (C =1 ): 95% Accuracy, 93.33% Sensitivity, 100% Specificity, 96% F1-score.

Bold value represents the best performance.

We evaluated the performance of the features using a linear kernel, a radial basis function (RBF), and a polynomial kernel (degree 3) kernel with SVM. Upon examining the overall performance across all metrics, it is evident that the wavelet-based features (Khandoker et al., 2007) consistently underperformed in the three classification approaches, regardless of the SVM kernel used. On the contrary, the proposed spectral features outperformed the features based on histograms and Poincaré plots (Begg et al., 2005) and wavelets (Khandoker et al., 2007).

In addition, the AUCs for wavelet-based features were poor, indicating that the classifier struggled to establish appropriate boundaries for these features. In contrast, both the spectral and histogram-Poincaré features yielded comparable AUC values. On multiple occasions, we observed a specificity of 100% for all three types of features (histogram features, Poincaré features, and the proposed spectral features).

### 4.3 Performance of proposed feature with different classifiers

Table 6 provides an overview of the performance achieved by five machine learning models after fine-tuning their hyperparameters.

**TABLE 6:** Classification performance metrics of spectral feature with three different cross-validation approaches using five classifiers.

Classifier	Approach											
	Leave-one-sample-out				Leave-one-fold-out				Five-fold-stratified-cross-validation			
	ACC	SENS	SPEC	F1	ACC	SENS	SPEC	F1	ACC	SENS	SPEC	F1
SVM	94.73	92.86	100.00	96.29	88.18	87.60	94.00	92.95	95.00	93.33	100.00	96.00
ANN	73.68	100.00	0.00	84.85	90.91	100.00	0.00	95.24	73.33	100.00	0.00	84.57
RF	89.47	92.85	80.00	92.85	87.27	87.00	90.00	92.39	95.00	93.33	100.00	96.00
EDT	94.73	92.85	100.00	96.29	61.45	64.00	36.00	60.95	90.00	93.33	80.00	93.14
AdaBoost	94.73	100.00	80.00	96.55	9.00	0.00	100.00	0.00	90.00	100.00	60.00	94.28

Using ACC and F1 as comparable metrics across three cross-validation approaches, SVM and RF classifiers using the proposed spectral features consistently perform well.

Metrics: Accuracy (ACC), Sensitivity (SENS), Specificity (SPEC), and F1-score (F1).

We experimented with two key hyperparameters for RF and EDT: minimum leaf size (1, 5, and 10) and the number of trees (randomly chosen between 5 and 100). In the case of the AdaBoost classifier, we focused on the learning rate (0.001, 0.01, and 0.1) and the number of weak learners (15, 20, 25, and 30). Regarding the artificial neural network (ANN), we varied the learning rate (0.1, 0.01, and 0.001) and the number of hidden nodes (10, 20, and 50) within a single hidden layer. We also performed hyperparameter optimization for the ANN, including optimization functions (Gradient Descent Backpropagation, Fletcher-Reeves Conjugate Gradient Descent, Polak-Ribiére Conjugate Gradient Descent), transfer functions (tan-sigmoid and log-sigmoid), a fixed number of epochs (1,000), and a ridge regularization value of 0.01.

Due to the limited sample size mentioned earlier in the classification section, we employed three cross-validation approaches to assess the effectiveness of these features.

#### 4.3.1 Leave-one-sample-out approach

We observed that SVM, EDT, and AdaBoost classifiers achieved the best performance, each with an F1-score of over 96%, and an accuracy of 94.73%. Notably, AdaBoost achieved 100% sensitivity, while SVM achieved 100% specificity. RF also performed well with an F1-score of 92.85%. However, ANN exhibited poor performance with a specificity of 0%.

#### 4.3.2 Leave-one-fold-out method

The SVM classifier demonstrated the best performance, achieving an F1-score of 92.95%, an accuracy of 88.18%, a specificity of 94.00%, and a sensitivity of 87.60%. RF also performed well with an F1-score of 92.39%. Conversely, ANN, EDT, and AdaBoost classifiers performed poorly in this scenario. The limited number of training samples in the leave-one-fold-out approach (only four samples from each class) may have hindered the ability of ANN, AdaBoost, and EDT to differentiate between the classes effectively.

#### 4.3.3 Five-fold stratified cross-validation

All classifiers performed reasonably well except for ANN. Both SVM and RF achieved the best performance with an F1-score of 96%. The poor performance of ANN across all these approaches can be attributed to its inability to train effectively with the limited number of samples available.

In summary, SVM demonstrated the best performance for most classification decision scenarios, while RF performance exceeded the other classifiers. Limited samples contributed to ANN’s poorer performance, and reduced training samples can also account for suboptimal discrimination using AdaBoost and EDT in the leave-one-fold-out method.

### 4.4 Influence of windowing

The presentation in Figure 6 and the statistical analysis Table 2 indicate that the three selected spectral features are equally powerful in distinguishing the two classes (*p* <0.001 , considering the entire database). In particular, the first component among these features corresponds to the DC value (magnitude at 0-th frequency bin) of the windowed MFC series. The DC value represents the average of a windowed segment, written as $\sum_{n=0}^{j-1}w\left(n\right)\cdot x\left(n\right)$ , where x(n) represents a frame of the MFC series, w(n) represents the window function, and j is the frame length.

The window function w(n) is multiplied element-wise with the corresponding samples of the frame x(n) and the resulting products are summed together to obtain the DC value for that window segment. Surprisingly, the average DC of all frames plays an equally important role in differentiating the two groups. Previous studies in the literature discussed using descriptive statistical features, including the average of the MFC series, as one of the features (Begg et al., 2005). However, windowing followed by calculating the mean allows for localized averages within each window, capturing more granular MFC fluctuations and proving more significant than the global mean of the entire MFC series.

### 4.5 Performance under noisy conditions

In real-world scenarios, foot clearance measurements obtained from stroke patients undergoing treadmill therapy can be subject to noise due to various factors. These factors include biological differences, such as variations in fitness levels and coping abilities, which can introduce stochasticity even among patients with similar lesions. Additionally, small and unpredictable fluctuations in the internal state, such as temporary increases in fatigue or shifts in effort and motivation, contribute to noise (Jin et al., 2022). On the external front, minor and unpredictable environmental disturbances introduce randomness into the measurements, including distractions, temperature variations, or gait perturbations caused by the moving treadmill belt. To this end, we evaluated the performance of the proposed spectral features and baseline features (Begg et al., 2005) in the presence of white Gaussian noise. We introduced noise to the test data while training the model on clean data. This evaluation helps us understand the robustness of the model and its features, considering that real-life test data can become noisy for various reasons. We added white Gaussian noise at different levels, ranging from 0% (representing clean data) to 10%, 20%, and 30% (representing increasing noise levels).

**FIGURE 7:**
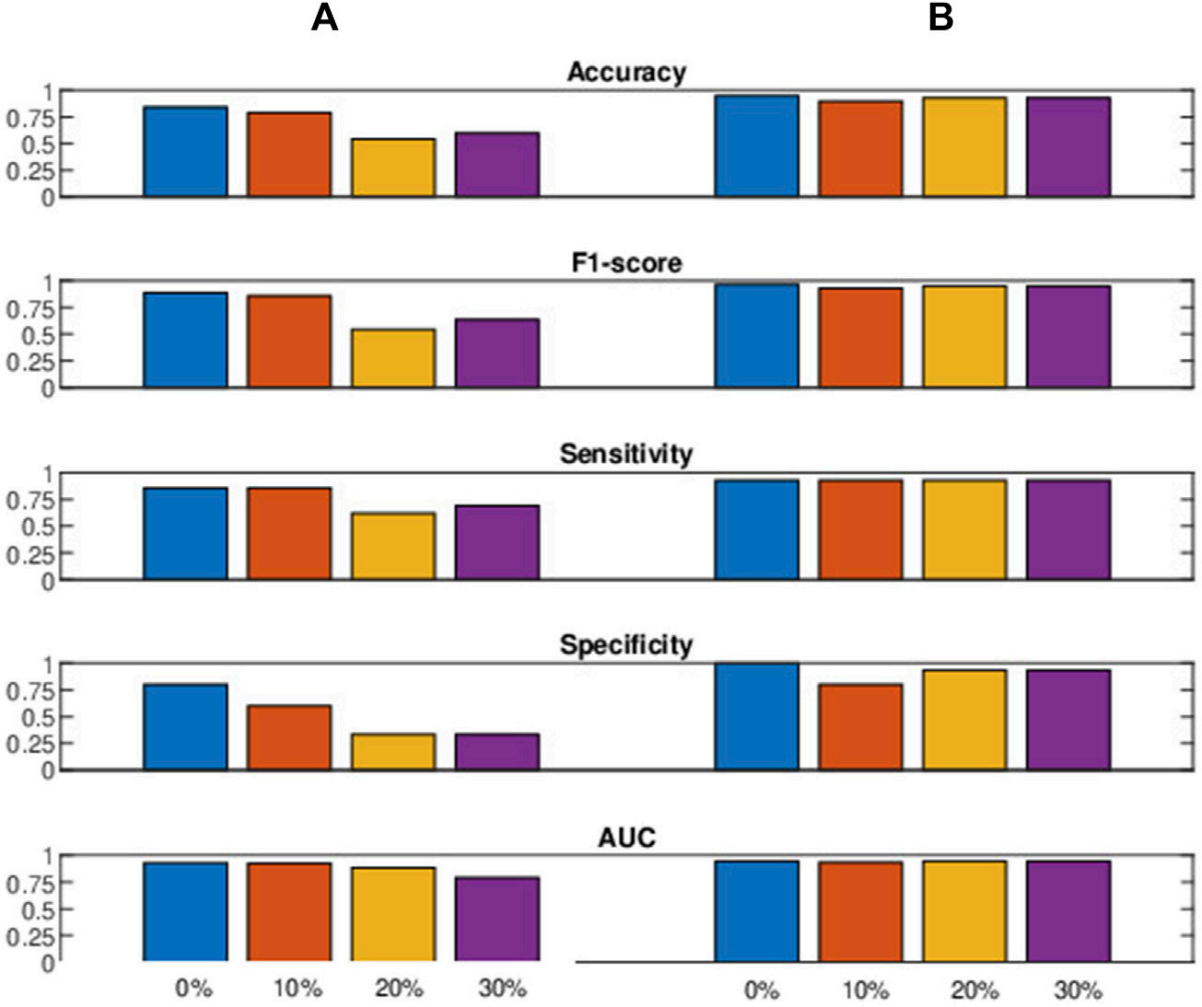
The figure presents performance metrics (decimal value: 0–1) with varying percentages of noise contamination in the test data, comparing the **(A)** baseline (Begg et al., 2005) with the **(B)** proposed spectral features.

Figure 7 presents the comparative results of cross-validating the leave-one-sample-out method using an SVM classifier. Spectral features exhibited greater robustness than histogram and Poincaré-based features (represented as baseline features) (Begg et al., 2005). The AUC values of the baseline features decreased as more noise was added to the test data, whereas the spectral features remained relatively robust across different percentages of noise. Interestingly, the spectral features declined abruptly, seen clearly in specificity, when 10% noise was introduced, possibly due to the regularization effect of noise contamination (Bishop, 1995). Considering the overall performance, we can conclude that the proposed spectral features outperformed the baseline features under noisy conditions.

## 5 Discussion

Accurately predicting the improvement in MFC after biofeedback training sessions based solely on their baseline data is of paramount importance in stroke rehabilitation. This predictive ability empowers healthcare professionals to create personalized treatment plans that are tailored to each individual’s needs. By identifying patients who are likely to benefit from such interventions, resources can be allocated more efficiently, optimizing patient outcomes while minimizing unnecessary expenditures. In this context, the MFC value serves as a widely recognized marker to determine the appropriate range of foot clearance during walking (Begg et al., 2019) to prevent falls and improve gait quality.

Considering the potential discomfort or limitations experienced by stroke patients when walking on a treadmill for extended periods, this study aimed to analyze MFC series data, encompassing approximately 200 strides. We aimed to predict the improvement in stroke patient’s condition, allowing targeted real-time biofeedback training for those who would benefit the most. The results indicate that using the MFC series and its frequency domain characteristics is crucial in achieving the desired objective. Furthermore, using features extracted from the frequency domain can contribute to developing a subject-independent model capable of automatically predicting patients who will experience improvement following the sessions.

The average spectrogram of the MFC values measured over multiple strides provides a characterization of the average frequency properties of MFC fluctuations. Averaging reduces variability and noise in the spectral estimates, highlighting the dominant frequencies across stride sequences, with mean spectrum peaks revealing rhythmic patterns linked to MFC fluctuations. The statistical analysis of the spectral features shown in Figure 6 and Table 2 reveals that the lower frequency values, including the DC component, were more influential in differentiating the improved and unimproved classes.

MFC values fluctuate rather than being strictly regular across strides. When we looked at its frequency spectrum in Figure 3, it showed a dominant cluster of lower frequencies. These lower frequencies reflected the underlying rhythmic dynamics that helped shape the fluctuations in the MFC series data. It is worth noting that a higher DC value in the spectrum indicated that the signal amplitudes exhibited minimal fluctuations over the strides. In the case of the unimproved class, the higher amplitude spectrum showed that the fluctuations across the strides were potentially more stable/sustained over the window (across strides) for the high-amplitude signal. On the contrary, in the case of the improving class, the MFC values were less stable.

**FIGURE 8:**
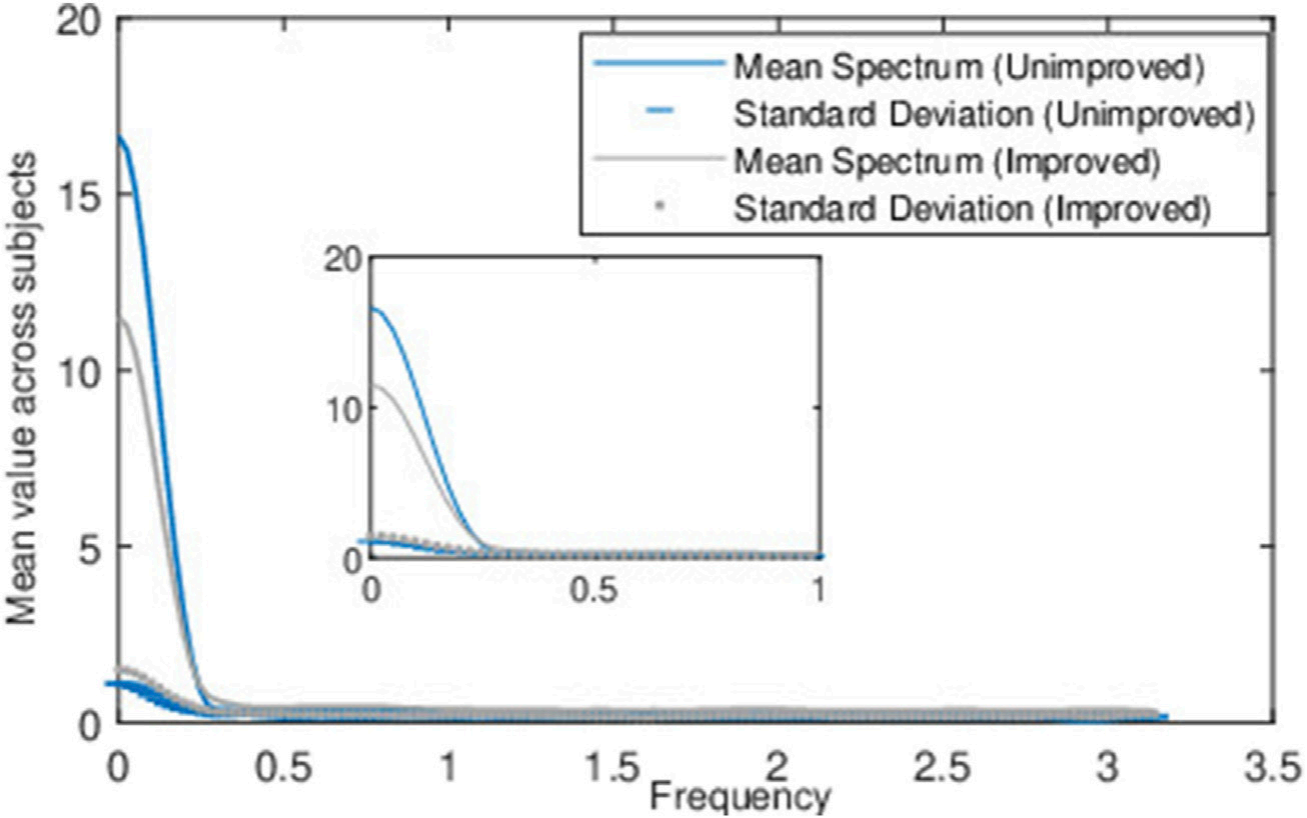
Figure represents the mean and standard deviation of magnitude spectrum across frames from the normalized baseline MFC data of all the stroke patients.

Figure 8 shows the mean magnitude spectrum of the MFC series across windows in all stroke patients, as well as the standard deviation for the improved and unimproved classes. The improved class shows more variance (or differences) in their frequency spectrum across windows compared to the unimproved class. This meant that the dominant frequencies representing the oscillation in the MFC values across the strides changed more and were less consistent. The unimproved class had less variance, and their dominant frequencies stayed more similar to the MFC values across strides. A more dominant mean frequency component and lower variation in MFC values between strides indicate a tendency to maintain a consistent stable pattern, potentially suggesting a lesser likelihood of improvement in the future.

The target band of biofeedback training was designed to increase and maintain the MFC within a specific range, aiming for an elevated MFC value and reduced variation in MFC across strides compared to the baseline. For patients exhibiting comparatively lower MFC values and higher fluctuations across strides (see, Mean, STD Feature of Table 2 and Figure 8), biofeedback training is found to be more effective in stabilizing their MFC values by increasing the mean value and reducing fluctuations. Conversely, when patients already have a relatively higher mean MFC and lower fluctuations, there is limited scope for enhancing the mean and decreasing fluctuations. This indicates that individuals who are more susceptible to tripping, characterized by lower MFC and greater variability in MFC (Sadeghi et al., 2000; Said et al., 2014), are likely to achieve superior outcomes with biofeedback training within this targeted range compared to less at risk of tripping, i.e., who possess higher MFC values and less variability. Regarding the second category of stroke patients, these individuals might benefit from additional training sessions or alternative target strategies to achieve successful outcomes. Another factor to consider could be the potential presence of spasticity in stroke patients (Park et al., 2021) whose MFC did not improve after completing 10 sessions of biofeedback training, indicating a promising area for exploration in future studies.

Table 2 demonstrates the superiority of window-based spectral features. In previous work, we used linear descriptive statistical features (Khandoker et al., 2016) in older adults’ MFC data, which were calculated from the entire MFC series without considering the non-stationarity of the MFC series. Tone and entropy features were computed from the time series of the percentage index, considering deviations between consecutive MFC data points. In the present dataset, to address non-stationarity, we have used windowing technique comprising more than two samples. Findings presented Tables 3–5 demonstrate the improved performance in stroke patients’ MFC data using short-term spectral features compared to features based on the histogram, Poincaré, and wavelet.

Although the wavelet transform is generally suitable for analyzing biological signals with various frequency components, it should be noted that in this study, the wavelet-based features (Khandoker et al., 2007) did not perform well. It is quite prominent that the variation of MFC values might be much higher in the case of older people than in the case of younger people (Khandoker et al., 2007). The multiscale exponent of the MFC signals, which captures correlations among variances of wavelet coefficients at different scales (multiresolution), proved more effective for that purpose (young vs. old) (Khandoker et al., 2007). However, in the context of stroke patients, the variation among different scales may not be as pronounced as that observed between younger and older individuals.

The proposed features showed consistent performance across most classifiers, except for the leave-one-fold-out ensemble decision tree and AdaBoost (refer to Table 6). This could be attributed to the limited number of samples for training (four per class) of these classifiers. Limited diversity and representation in low sample sizes might lead to overfitting, causing poor performance in ensemble decision tree and AdaBoost, whereas Random Forests’ additional randomization might mitigate these issues, resulting in better results. SVMs might perform well in lower sample sizes with linear, RBF, and polynomial kernels due to their margin-based optimization and regularization. In addition, the lower performance observed in the ANN across each of the cross-validation approaches is likely to be attributed to the smaller sample size creating overfitting. The results indicate that the proposed spectral feature demonstrated good performance with most classifiers. In some cases, the features based on the histogram and Poincaré performed equivalent to the proposed spectral feature. However, regardless of the classifier used, the wavelet-based feature consistently exhibited lower performance compared to the other two features.

## 6 Conclusion

In this article, novel features using the Short-term Fourier Transform (STFT)-based magnitude spectrum of MFC data are used to predict the effectiveness of biofeedback training. With our proposed approach, we can predict the effectiveness of real-time biofeedback training for a group of stroke patients solely based on their baseline data. The study revealed that short-term spectral components and the windowed mean value (DC value) carry significant information to predict the success of biofeedback training. The findings indicate that patients with high spectral amplitude and low variance in the lower frequency zone are less likely to show improvement, whereas patients with comparatively low spectral amplitude and high variance are more likely to show improvement after training.

Future research will focus on measuring changes after each training session for individual patients and identifying patterns of change specifically for those who show improvement after ten sessions, compared to those who do not. Applying long short-term memory (LSTM) neural networks (Zaroug et al., 2020) to predict kinematics of lower limb trajectories during biofeedback training would be useful to forecast MFC changes following biofeedback training session. Additionally, we plan to create a large database of patients to further evaluate our proposed features.

## Data Availability

The raw data supporting the conclusions of this article will be made available by the authors, without undue reservation.

## Footnotes

1
https://www.aihw.gov.au/reports/heart-stroke-vascular-disease/hsvd-facts/contents/all-heart-stroke-and-vascular-disease/stroke
